# The Application of Software “Rapid Processing of Perfusion and Diffusion” in Acute Ischemic Stroke

**DOI:** 10.3390/brainsci12111451

**Published:** 2022-10-27

**Authors:** Yudi Zhang, Shuang Song, Zhenzhong Li, Boyuan Huang, Yanlu Geng, Lihong Zhang

**Affiliations:** 1 Department of Neurology, The Second Hospital of Hebei Medical University, Shijiazhuang 050000, China; 2 Department of Radiology, The Second Hospital of Hebei Medical University, Shijiazhuang 050000, China; 3 Department of Neurology, Central Hospital of Qinghe County, Xingtai 054800, China

**Keywords:** RAPID software, PWI, CTP, acute ischemic stroke

## Abstract

In the event of an acute ischemic stroke, saving the penumbra is the most important aspect of early treatment. The rapid and accurate identification of ischemic penumbra plays a key role in its comprehensive treatment. At present, the identification method and evaluation standard of ischemic penumbra have not been unified. Numerous pieces of software identifying ischemic penumbra have been developed, such as rapid processing of perfusion and diffusion (RAPID), Sphere, Vitrea, and computed tomography perfusion+ (CTP+). The RAPID software, analyzing and integrating multi-mode image data (mainly based on perfusion weighted imaging (PWI) or computed tomography perfusion (CTP) images, shows good performance in identifying ischemic penumbra and has been utilized for the assessment of ischemic penumbra in many ischemic stroke clinical studies, achieving good outcomes and promoting the transition from “time window” to “tissue window” in the treatment of early stage AIS. To obtain a comprehensive understanding of the RAPID software and its accuracy in evaluating ischemic penumbra, this paper reviews the background and development of the RAPID software, summarizes the published acute cerebral infarction trials using the RAPID software, generalizes the threshold parameters in different time windows, and further discusses its application and limitations.

## 1. Introduction

Acute strokes pose the greatest threat to human health. They are characterized by high morbidity, disability, death, and recurrence rates. Acute ischemic stroke (AIS) is the most common subtype of acute stroke characterized by the reversible or irreversible loss of cerebral tissue and neurological function caused by an inadequate supply of cerebral blood flow [1]. In 2019, the global incidence of stroke was about 12.2 million, which is still the second leading cause of death worldwide, and the patients tend to be young; among people younger than 70 years old, the prevalence increased by 22.0% and the incidence increased by 15.0% [2]. The treatment of AIS involves recanalizing blood vessels through intravenous thrombolytic and/or intravascular therapy to salvage the ischemic penumbra in a transient time window. Ischemic penumbra refers to a fraction of brain tissues in the blood supply area of the occluded blood vessels, where cerebral blood flow is decreased and oxygen uptake is increased. Following the restoration of blood perfusion, oxygen metabolism and the nerve function of the brain tissues can be restored [3]. The gold standard for judging ischemic penumbra is positron emission tomography (PET) [3]. However, the technique is expensive, exposes the patient to high doses of radioactive material, and takes a longer amount of time to process the image data, which does not meet the need for the rapid treatment of acute cerebral infarction and restricts the wide and timely application of PET in the clinical practice of AIS. With the development of imaging technology, the assessment of ischemic penumbra has been replaced by computed tomography (CT) or magnetic resonance imaging (MRI), which utilizes the volume mismatch between the hypoperfused and infarct areas in the brain tissue. Because of its rapid feasibility and widespread availability, the MRI mismatch between perfusion- and diffusion-weighted imaging, as well as computed tomography perfusion, have been proposed as surrogate markers to identify the penumbra in AIS patients. How to quickly and accurately evaluate the mismatch zone and then determine the range of ischemic penumbra is of great significance for the transition from time window to tissue window in the treatment of early stage AIS, as well as the formulation of a treatment plan and the evaluation of prognosis. To date, numerous softwares identifying ischemic penumbra have been developed, such as RAPID, Sphere, Vitrea, and CTP+. However, at present, the judgment method and evaluation standard of the mismatched zone of hypoperfusion/infarction have not been unified, and the best parameters of the software for identifying the penumbra should be explored, tested, and validated in many clinical trials. As RAPID is approved by the Food And Drug Administration (FDA), it has been approved in more than 100 countries and used in more than 2000 hospitals worldwide (https://www.rapidai.com/, accessed on 28 September 2022). Moreover, in recent years, the RAPID software has been proved and applied in many international clinical studies to provide the software with a good chance for optimizing an appropriate parameter to identify penumbra. The current paper reviews the background, development, application, and limitation of the RAPID software and summarizes the history of clinical trials using RAPID to identify penumbra.

## 2. Development and Application of RAPID Software

### 2.1. Background and Development of RAPID Software

In 2006, the diffusion and perfusion imaging evaluation for understanding stroke evolution (DEFUSE) study [4], based on the algorithm of Ostergaard et al. [5,6] from the University of California, United States, developed and used an in-house software that utilized PWI to evaluate hypoperfused areas and diffusion weighted imaging (DWI) to evaluate infarction areas Appendix A. When processing PWI, firstly, the time-to-maximum (Tmax) map was built using the data in the arterial input, contralateral to an ischemic lesion, and obtained from the proximal middle cerebral artery. After a delay of two seconds at the ipsilateral side of the lesion was determined, the lower threshold for the hypoperfusion of the brain was also determined. Then, using a delay of 2 s, the Tmax map was used to identify the hypoperfused areas (PWI lesions), of which the volume was also calculated. When processing the DWI, the DWI lesion volume was calculated using the semi-automatic thresholding method. The DWI lesion area was defined as the area in which the DWI signal intensity was exceeded by more than three standard deviations of the corresponding normal brain tissue in the contralateral hemisphere, and the apparent diffusion coefficient (ADC) map was generated to confirm the DWI lesion as a new ischemic lesion. A PWI lesion with a volume greater than 10 mL and 120% of a DWI lesion was defined as a mismatch profile between perfusion and diffusion. In the 3 to 6 h follow-up PWI scan, there was an absolute reduction of more than 10 mL, or a relative reduction of more than 30% in the PWI lesion volume, which was indicative of early reperfusion. In the current study, 74 patients suffering from AIS were enrolled and, when the perfusion/diffusion profiles were mismatched at the baseline, early reperfusion was associated with improved clinical outcomes, establishing a threshold basis for RAPID. In 2010, Matus Straka et al. [7] developed a perfusion and diffusion image rapid processing software program (RAPID) that could automatically outline and calculate the lesion volume on PWI and DWI scans. It was used for operator-independent and automatic processing of image data, and all calculations could be completed within 5–7 min. The clinical evaluation speed of the ischemic penumbra was greatly accelerated. The software automatically identified the mismatch between the PWI and DWI scans using the segmentation of the Tmax map and infarct core of the ADC map. The present study analyzed the data of 74 AIS patients in the DEFUSE test [4], in which 63 patients were automatically identified and matched by the RAPID software. For the perfusion/diffusion mismatch detection, RAPID presented 100% sensitivity and 91% specificity compared with manual identification.

In 2011, Lansberg MG et al. [8] continued to review and analyze a total of 174 patients from the DEFUSE and echoplanar imaging thrombolytic evaluation (EPITHET) trials using the RAPID software, and observed that the patients with target mismatches (PWI_Tmax>6s_ lesion volume/DWI lesion volume > 1.2, DWI or PWI_Tmax>8s_ lesion ≤ 100 mL, and |PWI_Tmax>6s_ lesion volume − DWI lesion volume| ≥ 10 mL) achieved a better clinical prognosis following intravenous tissue plasminogen activator (tPA) reperfusion therapy, which could slow down the increase in infarction volume.

In 2011, the DEFUSE 2 study defined the ischemic core as ADC < 600 × 10^−6^ mm^2^/s and critically hypoperfused tissue as Tmax in PWI > 6 s. The criteria for identifying target mismatches were as follows: critically hypoperfused tissue/ischemic core ≥ 1.8; a |critically hypoperfused tissue − ischemic core| ≥ 15 mL; ischemic core volume < 70 mL; and lesion volume _Tmax>10s_ ≤ 100 mL. Among a total of 138 enrolled patients, 104 patients had reliable MR image quality (MRI profile cohort) and the consistent rate between automatic analysis by the RAPID software and the manual identification of lesion range reached 97% (101/104), which proved that the RAPID software accurately identifies hypoperfused and ischemic lesions and their locations [9]. A total of 59% (46/78) of patients with target mismatch and who received intravascular therapy experienced reperfusion, which was close to the percentage for patients without target mismatches (57%, 12/21). However, the recanalized patients with target mismatch achieved a better clinical outcome at 90 days than the recanalized patients without target mismatches.

In 2013, the RAPID software obtained the FDA 510 K clinical use license and is, at present, a medical image processing software package approved by the FDA. In 2015, the extending the time for thrombolysis in emergency neurological deficits (EXTEND) study confirmed that the automatic analysis of CTP-based hypoperfusion/infarction mismatches by the RAPID software was also rapid and reliable, and the processing time of RAPID was quicker with CTP data than MR data [10].

In 2017, several large trials of acute cerebral infarction successively used the RAPID software to select patients with target mismatches, and further explored the threshold parameter adjustment of the software in different time windows. The timeline of the main RAPID-related clinical trials is summarized in Table 1.

### 2.2. Application of RAPID Software

RAPID has been used as an image processing platform for hypoperfusion/infarction mismatches in large clinical studies, such as DEFUSE1, 2, 3; DAWN; SWIFT PRIME; EXTEND; and EXTEND-IA using MR or CT perfusion images.

When the Tmax threshold is 6 s, it is the most accurate value used to determine the hypoperfused area based on the voxel [7,9]. Recent studies have shown good consistency between MR and CT perfusion images in the recognition of lesions with Tmax > 6 s [15,16]. The threshold applied by RAPID was Tmax > 6 s and the relatively strict threshold was selected for at least two reasons. First, in RAPID, a shorter Tmax threshold resulted in artifacts within PWI lesions, affecting RAPID’s ability to automatically segment PWI lesions. Second, longer and more restrictive Tmax thresholds may reduce estimates of the extent of benign hypoperfusion [17].

Based on CTP, when the rCBF threshold was 0.30–0.34, the predicted infarct range presented the greatest consistency with the infarct range 27 h later [14]. The MR-based infarct area was defined as ADC 600 × 10^−2^ mm^2^/s, which was most consistent with the literature data [18,19]. In order to ensure the robustness of segmentation, the lesion with a volume < 1 mL was removed in the lesion mask [7]. An ADC lower than 560 × 10^−2^ mm^2^/s has a low segmentation sensitivity and an ADC higher than 640 × 10^−2^ mm^2^/s has a low segmentation specificity [7].

The DEFUSE3 study [12] enrolled 476 patients with anterior circulation AIS within 6–16 h of onset and RAPID software was used to prospectively establish continuous cohort MR and CT perfusion images receiving endovascular therapy. The target mismatch profile of MR or CT perfusion imaging in the current study was defined as a mismatch ratio ≥ 1.8, absolute volume of mismatch ≥ 15 mL, and infarct area less than 70 mL. Patients with a target mismatch assessed by RAPID also achieved good outcomes following endovascular treatment.

DAWN study [13] defined the mismatching between the infarct areas and NIH stroke scale (NIHSS) score, and patients undergoing intracranial internal carotid artery occlusion or M1 middle cerebral artery occlusion were selected. Within 6–24 h of the last normal time, rCBF images of DWI or CTP were automatically analyzed by RAPID software and, among the patients, three groups were formed: age ≥ 80 years, infarction area 0–21 cm^3^, and NIHSS ≥ 10; age < 80 years, infarction area 0–31 cm^3^, and NIHSS ≥ 10; and age < 80 years, infarction area 31–51 cm^3^, and NIHSS ≥ 20. The results show that patients with anterior circulation AIS with a small infarct size and mismatched clinical function scores can still take advantage of endovascular therapy within 6–24 h of the last normal time.

In SWIFT PRIME study [14,20], patients with an infarction volume ≤ 50 mL on MRI or CT scans, lesions with Tmax > 10 s on perfusion imaging ≤ 100 mL, mismatched volume ≥ 15 mL, and mismatched ratio > 1.8 were selected. The RAPID software was used to generate the initial baseline infarct area and lesion volume with Tmax > 6 s on perfusion imaging. The threshold for predicting the infarct area volume was set as a rCBF decrease of 70% compared with normal tissue [21], and the infarct core was set as ADC < 620 × 10^−6^ mm/s [4], which proved that, for patients who met the target mismatch value, the baseline lesion volume of the infarct area and initial perfusion imaging Tmax > 6 s was correlated with the infarct volume 27 h later [16]. Within 4.5 h of onset, rt-PA thrombolysis was administered intravenously in patients with anterior circulation AIS who met with the above conditions, and the treatment bridged with mechanical thrombectomy within 6 h of onset improved their prognosis 90 days following disease onset [20].

The EXTEND study [22] was designed to clarify whether the intravenous thrombolysis window could be extended to 9 h when multimodal imaging suggested a mismatch (absolute volume difference of more than 10 mL, ischemic core volume < 70 mL, and ratio between hypoperfusion area and ischemic core of more than 1.2). A total of 225 patients were included in the study. RAPID software was used for automatic analysis: (i) the hypoperfused area was defined as Tmax > 6 s of CTP or PWI; (ii) the volume of irreversibly injured ischemic core tissue was defined as rCBF < 30% of normal tissue for patients undergoing a CTP scan, and ADC ≤ 620 × 10^−6^ mm^2^/s for patients undergoing an MR scan [23]. The results show that, although the neurological deficits of the patients conforming to the above conditions improve following thrombolytic therapy, the proportion of symptomatic intracranial hemorrhage increases.

The EXTEND-IA study [11] determined that one of the reasons for the failure of the MRRESCUE experiment was that a reasonable and efficient image processing platform was not used, which affected the selection of target patients. This study confirmed that, in comparison with intravenous tPA alone, intravenous tPA thrombolysis with bridging artery thrombolysis improved ischemia reperfusion and early neurological functions in target patients within 4.5 h following onset. RAPID software was used to rapidly identify the hypoperfusion/infarction mismatch. For the automatic analysis of RAPID software, the mismatch definition was the same as the EXTEND study [22]. Subsequently, the team used RAPID software to further compare the thrombolytic therapy of tenaplase and alteplase prior to artery thrombolysis in patients with AIS using the same threshold value. As a result, tenaplase was superior to alteplase in the incidence of reperfusion and neurological prognosis prior to thrombolysis [24].

### 2.3. Advantages and Limitations of RAPID Software

The accurate estimation of the extent of the ischemic zone and infarct core area is the core problem and key purpose in the evaluation of ischemic penumbra. Compared with traditional image techniques, RAPID has apparent advantages. It only spends several minutes to evaluate an AIS patient, which could reduce the clinical decision time and improve the prognosis of AIS patients (demonstrated by the above-mentioned RCTs). RAPID also has good performance compared with other recently developed evaluation software. Rava et al. divided patients with AIS into endovascular and conservative treatment groups and compared the assessment of the ischemic zone and infarct core zone using RAPID, Sphere, Vitrea, and CTP+ software, respectively. The results show that, in the endovascular treatment group, RAPID has the least chance to overestimate the volume of the infarct core area, which presents patients with the greatest possibility for receiving endovascular treatment [25,26]. Nowadays, RAPID has been used in many hospitals and countries and in many clinical studies. Although RAPID’s rapid and accurate assessment of ischemic penumbra extended the time window for intravenous thrombolysis and endovascular therapy in AIS patients who meet certain criteria, and improved stroke outcomes, the practical application of RAPID in clinical practice is still not popular compared with traditional image techniques such as DWI. Moreover, the software has not been approved by government in all countries. For instance, in China, RAPID has yet to be formally used in clinical practice. Perhaps the leading reason is the lack of local RCT results and relatively high expenses. In addition, although the Chinese Guidelines for Standardized Application of Imaging in Cerebrovascular Disease (https://doi.org/10.3760/cma.j.issn.1005-1201.2019.11.002, accessed on 18 October 2022) and Chinese Stroke Association guidelines for clinical management of cerebrovascular disorders: executive summary and 2019 update of clinical management of ischaemic cerebrovascular diseases [27] suggest that, for patients with AIS, within 6–24 h of last known normal or if the the time of onset was unknown, who have large vessel occlusion in the anterior circulation, CTP or DWI and PWI are strongly recommended to evaluate the core infarct area and ischemic penumbra in patient selection for endovascular therapy. However, at present, CTP or PWI examination and endovascular treatment of acute ischemic stroke are mostly completed in advanced stroke centers, which are mostly concentrated in large and medium-sized cities; this may also limit the wide application of RAPID software in China. Additionally, some studies have shown that, for the evaluation of hypoperfusion/infarction mismatch, the consistency of the evaluation conclusions of multiple software is more practical than the absolute parameter setting of a single software [28]. Several points remain to be improved during the application of the RAPID software: (1) The estimation of the total volume of the hypoperfusion area in existing studies was based on the Tmax data of perfusion imaging, and whether the patients beyond the treatment time window are applicable has not been verified. (2) At present, the influence of various artifacts cannot be accurately excluded [13]. (3) Several large clinical trials using RAPID all targeted patients undergoing vascular recanalization, and the relevant threshold parameters may only apply to thrombolysis or thrombectomy. There are still gaps in the identification of mismatches in patients who exhibit mild or do not meet the criteria for thrombolysis or thrombectomy. Whether RAPID can also rapidly and accurately identify the perfusion and infarction mismatches in this kind of ischemic stroke remains to be explored further. (4) Most of the enrolled patients included in the current study suffered from anterior circulation occlusion, and the application value of the RAPID software to AIS patients with posterior circulation remains to be discussed.

## 3. Summary and Future Perspectives

This paper summarized the background, development, application, and limitations of the RAPID software in clinical research. Several studies have shown that RAPID software can rapidly and accurately judge the state of ischemic penumbra at the early stage of AIS, providing a histological basis for thrombolysis or thrombectomy. However, in the future, more clinical randomized controlled studies are needed to verify the evaluation power of RAPID software on ischemic penumbra and further optimize the “mismatch” parameter and its threshold, which may allow the software to be formally recommended by relevant guidelines and lay a foundation for extensive introduction into medical institutions and cost reduction. Moreover, it remains to be further explored whether RAPID can also accurately identify ischemic penumbra in most patients who have exceeded the time window for vascular recanalization. At present, our research team is exploring the use of RAPID software to evaluate the effects of butylphthalide (a national class A new drug in China used for AIS) [29,30,31] on the ischemic penumbral zone in patients with AIS beyond the time window of intravenous thrombolytic as well as intravascular therapy, so as to benefit more patients.

## Figures and Tables

**Table 1:** Application of RAPID software in various clinical trials.

Time	Researcher	Study	When to Apply Rapid Software	Parameters Set by Rapid Software			Conclusions
				Hypoperfusion	Ischemic Core	Mismatch	
2006 [4]	Albers GW et al.	DEFUSE	MRI scan was obtained 3 to 6 h after treatment with intravenous tPA 3 to 6 h after symptom onset	PWI _Tmax>2s_	Determined by the semi-automated thresholding method (exceeding the DWI signal intensity of the contralateral hemisphere by more than three standard deviations)	V_h_ ≥ 10 mL V_h_/V_i_ ≥ 1.2	For tPA-treated patients with a mismatch, especially target mismatch, there was a strong and highly significant association between early reperfusion and favorable clinical outcomes
2010 [7]	Straka M et al.	Review DEFUSE	Use RAPID software to analyze cases in DEFUSE	PWI _Tmax>6s_ V_P6_ ≥ 3 mL	ADC_th_: 600 × 10^−6^ mm^2^/s Vi ≥ 1 mL	V_h−_V_i_ ≥ 10 mL V_h_/V_i_ ≥ 1.2	Developed RAPID software, which can automatically segment and calculate lesion volume on PWI and DWI scans. The software has consistent results compared with a human reader
2011 [8]	Lansberg MG et al.	Review EPITHET and DEFUSE	Use RAPID software to analyze cases in EPITHET and DEFUSE (two studies of intravenous tPA administered in the 3 to 6 h time window).	PWI _Tmax>6s_ V_P6_ ≥ 10 mL V_P8_ ≤ 100 mL	10 mL ≤ V_i_ ≤ 100 mL	V_P6−_V_i_ ≥ 10 mL V_P6_/V_i_ ≥ 1.2	Comparing RAPID with processing methods used in previous stroke studies confirms that RAPID software can be used for patients with acute cerebral infarctions to rapidly select target patients
2012 [9]	Lansberg MG et al.	DEFUSE 2	Baseline MRI scan was obtained before treatment of patients. Endovascular treatment within 12 h of onset of stroke, followed by analysis with RAPID software to establish whether the patient has a mismatch	PWI _Tmax>6s_ V_P10_ ≤ 100 mL	ADC_th_: 600 × 10^−6^ mm^2^/s	V_h_/V_i_ ≥ 1.8 V_h−_V_i_ ≥ 15 mL V_i_ < 70 mL	For endovascular-treated patients with the target mismatches, there was a strong and highly significant association between early reperfusion and favorable clinical outcomes
2014 [11]	Campbell BC et al.	EXTEND-IA	Baseline MRI or multimodal CT scan was obtained before or immediately after treatment of patients commencing intravenous tPA within 4.5 h of onset of anterior circulation ischemic stroke, followed by analysis with RAPID software to establish whether the patient has a mismatch	CTP _Tmax>6s_ or PWI _Tmax>6s_	Diffusion lesion or rCBF decrease < 30% or ADC_th_: 620 × 10^−6^ mm^2^/s	V_h_/V_i_ > 1.2 V_h−_V_i_ > 10 mL V_i_ < 70 mL	For patients within a 4.5 h onset of anterior circulation ischemic stroke with the target mismatch, treatment with intra-arterial clot retrieval after intravenous tPA improved reperfusion and early nervous system compared with intravenous tPA alone
2015 [10]	Campbell BC et al.	part of the EXTEND (tPA/placebo 4.5–9 h poststroke) and EXTEND-IA (tPA < 4.5 h ± thrombectomy)	Patients presenting < 9 h from stroke had CT, CTP, or CTA, followed by analysis with RAPID software to establish whether the patient has a mismatch	CTP _Tmax>6s_	rCBF < 30%	V_h_/V_i_ > 1.2 V_h−_V_i_ > 10 mL V_i_ < 70 mL	CTP and perfusion–diffusion MRI data were processed using RAPID software to generate a ‘mismatch’ classification that determined eligibility for trial therapies
2017 [12]	Albers GW et al.	DEFUSE 3	Baseline MRI or multimodal CT scans were obtained before treatment of patients. Endovascular treatment within 6–16 h of onset of ICA or MCA occlusion, followed by analysis with RAPID software to establish whether the patient has a mismatch	PWI _Tmax>6s_ V_P10_ ≤ 100 mL	ADC_th_: 600 × 10^−6^ mm^2^/s	V_h_/V_i_ ≥ 1.8 V_h−_V_i_ ≥ 15 mL V_i_ < 70 mL	For patients with ICA or MCA occlusions and target mismatches on multimodal CT or MR imaging, endovascular therapy in a time window of 6 to 16 h may be beneficial
2017 [13]	Jovin TG et al.	DAWN	Baseline MRI or multimodal CT scan was obtained before treatment of patients. Endovascular treatment within 6–24 h from TLSW, followed by analysis with RAPID software to evaluate ischemic core size	Use of NIHSS as an indicator of tissue at risk in lieu of perfusion studies (CCM)	Age ≥ 80 y, V_i_ < 21 cm^3^; Age < 80 y, V_i_ < 31 cm^3^; Age < 80 y, 31 ≤ V_i_ ≤ 51 cm^3^	Age ≥ 80 year, NIHSS ≥ 10, V_i_ < 21 cm^3^; Age < 80 year, NIHSS ≥ 10, V_i_ < 31 cm^3^; Age < 80 year, NIHSS ≥ 20, 31 ≤ V_i_ ≤ 51 cm^3^	Compared with subjects treated with standard medical therapy alone, there were better outcomes and substantial areas of salvageable brain based on age-adjusted clinical core mismatches of patients who could experience endovascular treatment within 6–24 h from TLSW
2017 [14]	Mokin M	Review SWIFT PRIME	Use RAPID software to analyze cases of both intravenous tPA only and endovascular treatment in DEFUSE	V_P10_ ≤ 100 mL	rCBF < 30%	V_h_/V_i_ ≥ 1.8 V_h_-V_i_ ≥ 15 mL V_i_ ≤ 50 mL	The most accurate thresholds for predicting the final 27 h infarct volume were rCBF 0.30~0.34 or rCBV 0.32~0.34

V_h_: the volume of hypoperfused tissue; V_i_: the volume of the ischemic core; ADC_th_: ADC threshold; V_P6_: the volume of PWI _Tmax>6s_; V_P8_: the volume of PWI _Tmax>8s_; V_P10_: the volume of PWI _Tmax>10s_; rCBF: relative cerebral blood flow; rCBV: relative cerebral blood volume; TLSW: time last seen well; CTA: Computed tomography angiography; ICA:Internal Carotid Artery; MCA:Middle Cerebral Artery; EXTEND-IA: Extending the time for thrombolysis in emergency neurological deficits with intra-arterial therapy; DAWN: DWI or CTP assessments with clinical mismatches in the triage of wake-up and late-presenting strokes undergoing neuro-intervention with Trevo; SWIFT PRIME: solitaire with the intention for thrombectomy as the primary endovascular treatment.

**Figure A1:**
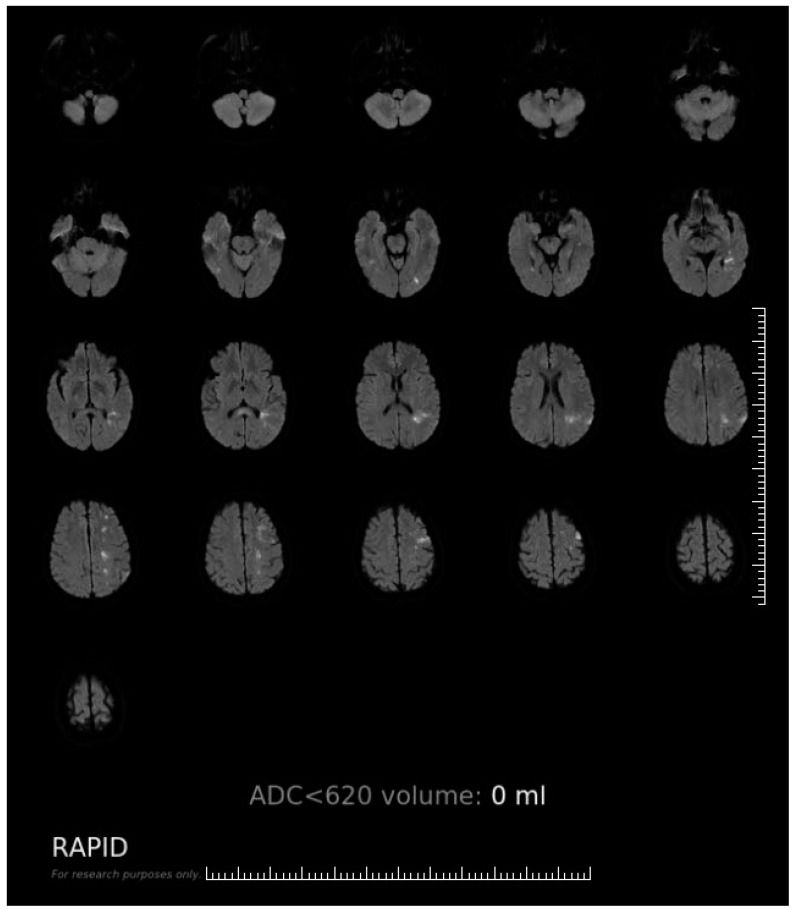
ADC map of a patient.

**Figure A2:**
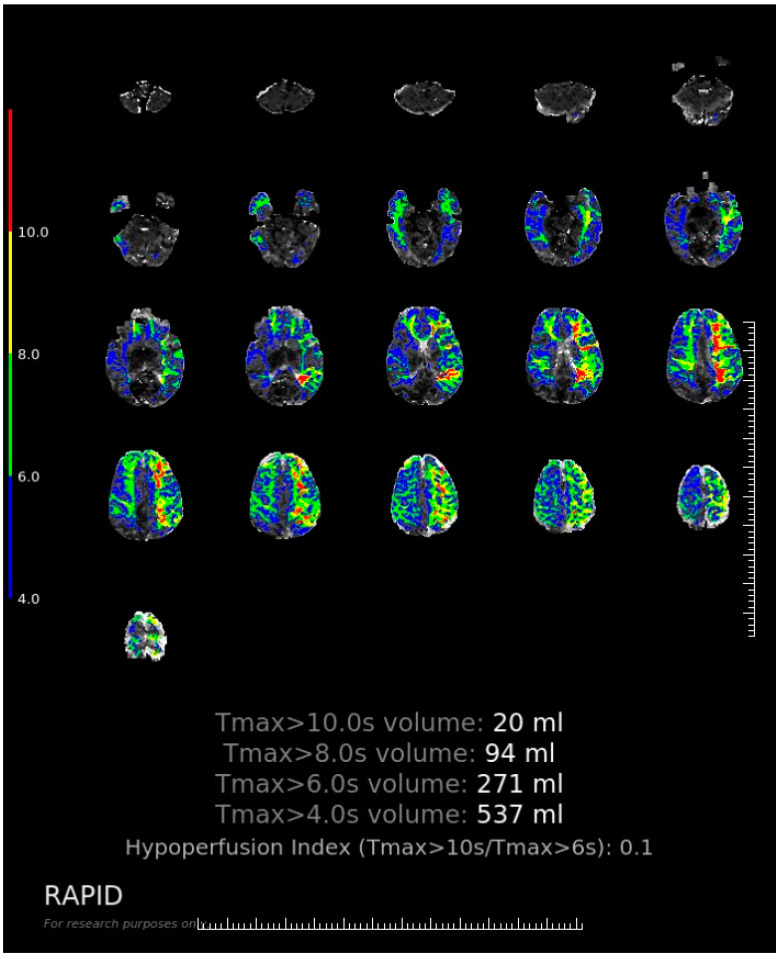
The hypoperfused area calculated by the Tmax map.

**Figure A3:**
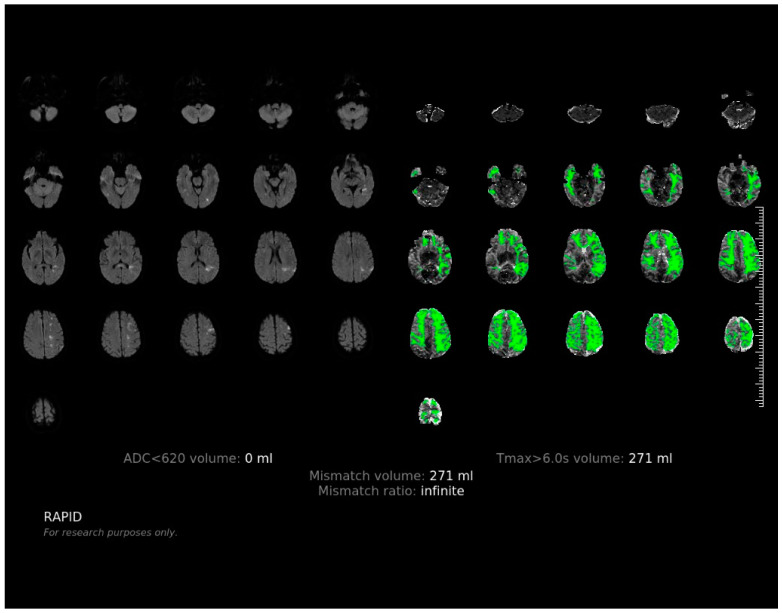
The calculation of the mismatch area.

## Appendix A. The Software Interface and Evaluating Procedure

Step 1: Calculating the range and volume of the ischemic core.

Using DWI to generate ADC map and plot the range of the ischemic core by the parameter ADC < 620 × 10^−2^ mm^2^/s. Taking Figure A1 as an example, although there are DWI-positive lesions in many cross sections of the brain, the volume of the ADC < 620 × 10^−2^ mm^2^/s area is 0; therefore, the ischemic core is 0.

Step 2: Calculating the hypoperfused area.

Using gadolinium-contrasted PWI or CTP images, Tmax map was created. The range and volume of the area (Tmax > 4 s, Tmax > 6 s, Tmax > 8 s, and Tmax > 10 s) were calculated. Among them, the Tmax > 6 s area was defined as the hypoperfused area, which presented the risk of rapid deterioration into brain infarction (Figure A2).

Step 3: Calculating the mismatch.

Calculate the volume difference between the hypoperfused area and ischemic core, which was defined as a mismatch (Figure A3).
